# Supplementary material for: An analytical delay model for multi-class and lane-free traffic condition
Source: PLoS One. 2025 Feb 21;20(2):e0319325. doi: 10.1371/journal.pone.0319325 (PMC11845045; doi:10.1371/journal.pone.0319325)
Supplement: Attachment — Submitted filename: Response to Reviewers.docx [file pone.0319325.s004.docx]

**PONE-D-24-24169**

**An Analytical Delay model for Multi class and Lane free Traffic condition**

We thank the reviewers and the editor for their constructive comments. We have provided our responses below and the same have been incorporated in the revised manuscript appropriately.

**Academic editor**

**Comment 1:**

Please review your reference list to ensure that it is complete and correct. If you have cited papers that have been retracted, please include the rationale for doing so in the manuscript text, or remove these references and replace them with relevant current references. Any changes to the reference list should be mentioned in the rebuttal letter that accompanies your revised manuscript. If you need to cite a retracted article, indicate the article’s retracted status in the References list and also include a citation and full reference for the retraction notice.

**Response:** Thank you for your feedback and for allowing us to submit the revised version of the manuscript. We have carefully reviewed the reference list to ensure its completeness and accuracy. We confirm that none of the cited papers have been found to be retracted. The revised reference list, adding DOIs, is given below. Please let us know if there is any concern about any specific papers.

1. Mousa RM, Asce M. Analysis and Modeling of Measured Delays at Isolated Signalized Intersections. J Transp Eng A Syst. 2002;128(4): 347-354. DOI 10.1061/ASCE0733-947X2002128:4347.
2. Lin C, Gong B, Qu X. Low Emissions and Delay Optimization for an Isolated Signalized Intersection Based on Vehicular Trajectories. PLoS ONE. 2015;10(12): e0146018. DOI: 10.1371/journal.pone.0146018.
3. Olszewski P. Overall Delay, Stopped Delay, and Stops at Signalized Intersections. J Transp Eng A Syst. 1993;119(6): 835-852. DOI: 10.1061/(ASCE)0733-947X(1993)119:6(835).
4. Mathew TV, Radhakrishnan P. Calibration of Microsimulation Models for Non lane Based Heterogeneous Traffic at Signalized Intersection. J Urban Plan Dev. 2010;136(1): 59-66. DOI: 10.1061/ASCE0733-94882010136:159.
5. Khadhir A, Maripini H, Sreedhar S, Vanajakshi L, Chilukuri BR. A Study of Delay Estimation Methods at Signalized Intersections for Mixed Traffic Condition. Transportation in Developing Economies. 2021;7: 1-16. DOI: 10.1007/s40890-021-00120-9.
6. Verma A, Nagaraja G, Anusha CS, Mayakuntla SK. Traffic Signal Timing Optimization for Heterogeneous Traffic Conditions Using Modified Webster’s Delay Model. Transportation in Developing Economies. 2018;4: 1-13. DOI: 10.1007/s40890-018-0064-2.
7. Preethi P, Varghese A, Ashalatha R. Modelling Delay at Signalized Intersections under Heterogeneous Traffic Conditions. Transportation Research Procedia. 2016;17: 529–538. DOI: 10.1016/J.TRPRO.2016.11.107.
8. Arasan VT, Jagadeesh K. Effect of Heterogeneity of Traffic on Delay at Signalized Intersections. J Transp Eng A Syst. 1995;121(5): 397–404. DOI: 10.1061/(ASCE)0733-947X(1995)121:5(397).
9. Fambro DB, Rouphail NM. Generalized Delay Model for Signalized Intersections and Arterial Streets. Transp Res Rec. 1997;1572(1): 112-121. DOI: 10.3141/1572-14.
10. Akgungor AP. A new delay parameter dependent on variable analysis periods at signalized intersections. Part 1: model development. Transport. 2008;23(1): 31–36. DOI: 10.3846/1648-4142.2008.23.31-36.
11. Ban XJ, Li Y, Skabardonis A, Margulici JD. Performance evaluation of travel-time estimation methods for real-time traffic applications. Journal of Intelligent Transportation Systems: Technology, Planning, and Operations. 2010;14(2): 54–67. DOI: 10.1080/15472451003719699.
12. Cheng Y, Qin X, Jin J, Ran B, Anderson J. Cycle-by-cycle queue length estimation for signalized intersections using sampled trajectory data. Transp Res Rec. 2011;2257(1): 87–94. DOI: 10.3141/2257-10.
13. Dion F, Rakha H, Kang YS. Comparison of delay estimates at under-saturated and over-saturated pre-timed signalized intersections. Transportation Research Part B: Methodological. 2004;38(2): 99–122. DOI: 10.1016/S0191-2615(03)00003-1.
14. Roess RP, McShane WR, Prassas ES. Traffic engineering. 2nd ed. Upper Saddle River, N.J: Prentice Hall, 1998.
15. Newell GF. Approximation Methods for Queues with Application to the Fixed-Cycle traffic Light. SIAM Rev Soc Ind Appl Math. 1965;7(2): 223-240. DOI: 10.1137/1007038.
16. May AD, Keller HEM. A deterministic queueing model. Transportation Research. 1967;1(2): 117–128. DOI: 10.1016/0041-1647(67)90167-0.
17. P Wagner, NH Gartner, T Lu, R Oertel. Webster’s Delay Formula- revisited. 93rd Annual meeting of Transportation Research Board, Washington. 2013.
18. Beckman M, McGuire CB, Winsten CB, Koopmans TC. Studies in the economics of transportation. New Haven: Yale University Press. Cowles Commission for Research in Economics. 1956;7(4), 146.
19. Newell GF. Queues for a ﬁxed-cycle traﬃc light. The Annals of Mathematical Statistics. 1960;31(3): 589–597. DOI:10.1214/aoms/1177705787.
20. Allsop RE. Delay-minimizing settings for ﬁxed-time traﬃc signals at a single road junction. IMA Journal of Applied Mathematics. 1971;8(2): 164–185. DOI: 10.1093/imamat/8.2.164.
21. Kendall DG. Some problems in the theory of queues. J R Stat Soc Series B Stat Methodol. 1951;13(2): 151–173. DOI: 10.1111/j.2517-6161.1951.tb00080.x.
22. Webster FV. Traffic Signal Settings. Road Research Technical Paper 39, Road Research Laboratory, London. 1958.
23. Miller AJ. Settings for Fixed-Cycle Traffic Signals. Operations Research. 1963;14(4): 373-386. DOI: 10.2307/3006800.
24. Mcneil DR. A Solution to the Fixed-Cycle Traffic Light Problem for Compound Poisson Arrivals. Source: Journal of Applied Probability. 1968;5(3): 624-635. DOI: 10.2307/3211926.
25. Heidemann D. Queue Length and Delay Distributions at Traffic Signals. Transportation Research Part B: Methodological. 1994;28(5): 377-389. DOI: 10.1016/0191-2615(94)90036-1.
26. Hutchinson TP. Delay at a Fixed Time Traffic Signal-2. Numerical Comparisons of Some Theoretical Expressions. Transportation Science. 1972;6(3): 286–305. DOI: 10.1287/trsc.6.3.286.
27. Catling I. A time-dependent approach to junction delays. Trafﬁc Engineering and Control. 1977;18(11): 520–526.
28. Akcelik R. Time-dependent expressions for delay, stop rate and queue length at traﬃc signals. Internal Report AIR 367(1). Australian Research Board. 1980.
29. Kimber R, Hollis E. Trafﬁc queues and delays at road junctions. TRRL Laboratory Report, 909, UK. 1979.
30. Transportation Research Board TRB. Highway capacity manual 1985, National Research Council, TRB, Washington, D.C. 1985.
31. Akçelik R. Traffic Signals: Capacity and Timing Analysis. Report ARR No.123. Australian Research Board. 1981.
32. Teply S, Allingham DI, Richardson DB, Stephenson BW. Canadian capacity guide for signalized intersections. 20081984.
33. Akçelik R. The Highway Capacity Manual delay formula for Signalised Intersections. ITE Journal. 1988;58(3): 23-27.
34. Burrow I. A note on traffic delay formulas. ITE Journal. 1989;59(10).
35. Transportation Research Board TRB. Highway capacity manual 2000, National Research Council, TRB, Washington, D.C. 2000.
36. Lee TC, Wong KI. An agent-based model for queue formation of powered two-wheelers in heterogeneous traffic. Physica A: Statistical Mechanics and its Applications. 2016;461: 199–216. DOI: 10.1016/j.physa.2016.05.005.
37. Kiran MS, Verma A. Review of studies on mixed trafﬁc ﬂow: Perspective of developing economies. Transportation in Developing Economies. 2016;2(1): 1-16. DOI: 10.1007/s40890-016-0010-0.
38. J Zhao, VL Knoop, J Sun, Z Ma, M Wang. Unprotected Left-Turn Behavior Model Capturing Path Variations at Intersections. IEEE trans Intell Transp Syst. 2023;24(9): 9016–9030. DOI: 10.1109/TITS.2023.3270962.
39. Hoque MS, Asif Imran M. Modification of Webster’s delay formula under non-lane based heterogeneous road traffic condition. Journal of Civil Engineering (IEB). 2007;35(2): 81-92.
40. Medhi, J. Stochastic models in queueing theory. Academic Press. 2002.
41. Mukhopadhyay S, Pramod MJ, Kumar A. Approximate Mean Delay Analysis for a Signalized Intersection With Indisciplined Traffic. IEEE trans Intell Transp Syst. 2017;18(10): 2750-2762. DOI: 10.1109/TITS.2017.2665121.
42. Khadhir A, Bhaskar A, Vanajakshi L, Haque MM. Development of a Theoretical Delay Model for Heterogeneous and Less Lane-Disciplined Traffic Conditions. J Adv Transp. 2022;2022(2): 1-20. DOI: 10.1155/2022/3260945.
43. Indo-HCM. Indian Highway Capacity Manual (Indo-HCM) 2017. CSIR-Central Road Research Institute, New Delhi. 2017.
44. PS Bokare, Akhilesh Kumar Maurya. Study of Acceleration Behaviour of Various Vehicle Types. Transportation Research Procedia, 2017;25: 4733–4749. DOI: 10.1016/j.trpro.2017.05.486.
45. Anand A, Ramadurai G, Vanajakshi L. Data fusion-based traffic density estimation and prediction. Journal of Intelligent Transportation Systems: Technology, Planning, and Operations. 2014;18(4): 367–378. DOI: 10.1080/15472450.2013.806844.
46. PTV Group. PTV VISSIM 11 USER MANUAL, no 1055. 2018.
47. Katkar R, Venugopal A, Saikrishna CA, Vanajakshi L. Calibration of Microscopic Traffic Simulation for Signalized Intersections Under Heterogeneous Traffic Conditions. Recent Advances in Traffic Engineering. RATE 2022. Lecture Notes in Civil Engineering; 377, Springer, Singapore. 2024. DOI: 10.1007/978-981-99-4464-4_22.
48. Siddharth SMP, Ramadurai G. Calibration of VISSIM for Indian Heterogeneous Traffic Conditions. Procedia Soc Behav Sci. 2013;104: 380–389. DOI: 10.1016/j.sbspro.2013.11.131.
49. Frank D. Simulation of a multi-station system of queues with delayed exponential service. Institute of statistics, Mimeograph series No. 541. 1967.
50. Raj P, Sivagnanasundaram K, Asaithambi G, Ravi Shankar AU. Review of Methods for Estimation of Passenger Car Unit Values of Vehicles. J Transp Eng A Syst. 2019;145(6): 04019019. DOI: 10.1061/jtepbs.0000234.
51. Sharma M, Biswas S. Estimation of Passenger Car Unit on urban roads: A literature review. International Journal of Transportation Science and Technology. Elsevier B.V. 2021;10(3): 283–298. DOI: 10.1016/j.ijtst.2020.07.002.
52. Ackoff RL, Sasieni MW. Fundamentals of Operations Research. India: Wiley Eastern. 1968.
53. Little JDC. A proof for the queuing formula: L=λW. Operations Research. 1961;9(3): 383–387. DOI: 10.1287/opre.9.3.383.

**Comment 2:**

The manuscript has been thoroughly revised. The authors would need to respond/address Reviewer 2's comments.

**Response:** Thank you for your feedback and for providing us the opportunity to revise our manuscript. We have thoroughly reviewed and addressed all of Reviewer 2's comments in the best possible way. The detailed responses can be found in the rebuttal letter, and the necessary revisions have been incorporated into the manuscript.

**Reviewer #1:**

I suggestions have been properly addressed. The current version can be published. I have no more comments.

**Response:** Thank you for your positive feedback and confirmation. We greatly appreciate the time and effort you have dedicated to reviewing our manuscript and for providing valuable insights.

**Reviewer #2:**

The authors did not address my comments properly. I requested that they enhance the literature review and clarify the equation explanations, but they did not comply.

**Response:** Thank you for your feedback. We apologize if our previous revisions did not fully meet your expectations. In response to the comments provided in the last review, we have added the following literature to enhance the review section earlier.

1. Roess RP, McShane WR, Prassas ES. Traffic engineering. 2nd ed. Upper Saddle River, N.J: Prentice Hall, 1998.
2. Newell GF. Approximation Methods for Queues with Application to the Fixed-Cycle traffic Light. SIAM Rev Soc Ind Appl Math. 1965;7(2): 223-240. DOI: 10.1137/1007038.
3. May AD, Keller HEM. A deterministic queueing model. Transportation Research. 1967;1(2): 117–128. DOI: 10.1016/0041-1647(67)90167-0.
4. Beckman M, McGuire CB, Winsten CB, Koopmans TC. Studies in the economics of transportation. New Haven: Yale University Press. Cowles Commission for Research in Economics. 1956;7(4), 146.
5. Newell GF. Queues for a ﬁxed-cycle traﬃc light. The Annals of Mathematical Statistics. 1960;31(3): 589–597. DOI:10.1214/aoms/1177705787.
6. Allsop RE. Delay-minimizing settings for ﬁxed-time traﬃc signals at a single road junction. IMA Journal of Applied Mathematics. 1971;8(2): 164–185. DOI: 10.1093/imamat/8.2.164.
7. Kendall DG. Some problems in the theory of queues. J R Stat Soc Series B Stat Methodol. 1951;13(2): 151–173. DOI: 10.1111/j.2517-6161.1951.tb00080.x.
8. Hutchinson TP. Delay at a Fixed Time Traffic Signal-2. Numerical Comparisons of Some Theoretical Expressions. Transportation Science. 1972;6(3): 286–305. DOI: 10.1287/trsc.6.3.286.
9. Catling I. A time-dependent approach to junction delays. Trafﬁc Engineering and Control. 1977;18(11): 520–526.
10. Akcelik R. Time-dependent expressions for delay, stop rate and queue length at traﬃc signals. Internal Report AIR 367(1). Australian Research Board. 1980.
11. Kimber R, Hollis E. Trafﬁc queues and delays at road junctions. TRRL Laboratory Report, 909, UK. 1979.
12. Transportation Research Board TRB. Highway capacity manual 1985, National Research Council, TRB, Washington, D.C. 1985.
13. Teply S, Allingham DI, Richardson DB, Stephenson BW. Canadian capacity guide for signalized intersections. 20081984.
14. Burrow I. A note on traffic delay formulas. ITE Journal. 1989;59(10).

Based on the reviewer’s comments, additional relevant literature specific to lane-free traffic conditions-based delay modeling has been reviewed and incorporated into the manuscript. The details are as follows:

1. Kiran MS, Verma A. Review of studies on mixed trafﬁc ﬂow: Perspective of developing economies. Transportation in Developing Economies. 2016;2(1): 1-16. DOI: 10.1007/s40890-016-0010-0.

The insights from this paper have been added to the last paragraph of the time-dependent delay section in the literature review. The revised text is as follows:

“Unlike conventional car following, gap filling emerges as the predominant behaviour in such scenarios [37]. The movement of vehicles can be affected by factors such as geometric design, signal control settings, driver behaviour and traffic conditions [38].”

2. Mukhopadhyay S, Pramod MJ, Kumar A. Approximate Mean Delay Analysis for a Signalized Intersection With Indisciplined Traffic. IEEE trans Intell Transp Syst. 2017;18(10): 2750-2762. DOI: 10.1109/TITS.2017.2665121.

The section on delay models for MCLF traffic conditions is updated with a review of this paper as follows:

“Mukhopadhyay et al. [41] extended Webster's mean delay formula to account for MCLF traffic conditions by incorporating a Markovian service process. However, their analysis assumes a single-server scenario, which does not accurately reflect the characteristics of MCLF traffic conditions.”

The equations have been clarified and thoroughly explained by discussing the derivation from the basics as:

“The fundamental structure of any theoretical delay model includes a uniform delay term, random delay term, overflow delay term, and adjustment factors that are typically site-specific. The current formulation of the delay model also follows the same structure. However, this study assumes that the intersection is under-saturated, and hence, the overflow delay term, which accounts for delays caused by flow exceeding the capacity, is omitted.

The first term, known as the uniform delay term, is derived from the cumulative arrival and departure curves. This term represents the deterministic component of delay, as illustrated in Fig 9. In this context, the area between the cumulative arrivals and departures corresponds to the total delay experienced by all vehicles seeking to traverse through the intersection in a single cycle. Equation (5) can then be derived to calculate the average uniform delay (*d_1_*) with the assumption of uniform arrivals.

**Fig 9. Deterministic component of delay**

$d_{1}=\frac{C\left( 1-\frac{g}{C} \right)^{2}}{2\left( 1-\frac{q}{s} \right)}.$	(5)Substituting *λ=g/C* and *X=q/(sg/C),* Equation (5) becomes:

$d_{1}=\frac{C\left( 1-\lambda\right)^{2}}{2\left( 1-\lambda X \right)}.$	(6)

The second term corresponds to the random delay, which accounts for the random fluctuations in the arrivals. In this condition, some cycles may fail, even though the whole intersection *q/c* is less than one. Fig 10 illustrates the random delay, where dotted line represents the capacity. Area between the arrival curve and this dotted line depicts the random delay. In traditional models, this term is derived assuming random arrivals and deterministic departures. However, under MCLF conditions, the arrivals and departures follow a random distribution. Incorporating all these, the delay expression was derived as discussed below.

**Fig 10. Random component of delay**

As discussed earlier, a multi-channel queueing process is considered as the best way to represent the queue formation under MCLF traffic conditions. As the arrivals and departures are stochastic under MCLF traffic condition, the system is modelled as M/M/n system, which follows Poisson arrivals and departure with *n* service channels. The corresponding random delay term (*d_2_*) is derived based on this.

The basic derivation starts with the concept of birth-death process. One of the basic assumptions made in earlier studies [40] is the system is always operating at its full capacity. This assumption is not realistic for signalized intersection, as discharge occur only during green signals. Consequently, capacity is a more appropriate term for describing departure from a signalized intersection. Using this concept, for a multiple channel queueing system, there are four conditions at time *t* which must be considered in the formulation of transient equations, which are:

1. The system is empty with no entities (*N*=0).
2. The number of entities is less than the number parallel servers (*N<n*)*.*
3. The system with number of entities and number of parallel servers are same (*N=n*).
4. The number of entities is higher than the number of parallel servers (*N>n*).

Let $P_{0}\left( t+\Delta t \right)$ and $P_{N}\left( t+\Delta t \right)$ be the probability that the queueing system has zero and *N* entities at the time $+\Delta t$ . There are three ways in which the system could have reached this state, assuming $\Delta t$ is so small that only one unit could have entered or departed.

a) The system did not change from time t to $t+\Delta t$
b) The system was in state *N-1* at *t*, and one arrived in $\Delta t$
c) The system was in state *N+1* at *t*, and one departed in $\Delta t$

Since the probability of one arrival in $\Delta t$ equals $q\Delta t$ and the probability of one departure in $\Delta t$ is $c\Delta t$, the corresponding probabilities of zero arrivals and zero departures are *1 -* $q\Delta t$ and *1-*$c\Delta t$. If higher orders of $\Delta t$ are neglected, the mathematical description of the three ways for the system to reach *N* and zero entities in $t+\Delta t$ of the above-stated four conditions of multiple channel queueing system are:

$P_{0}\left( t+\Delta t \right)=P_{0}\left( t \right)\left( 1-q\Delta t \right)+P_{1}\left( t \right)\left( 1-q\Delta t \right)c\Delta t.$	(7)

$P_{N}\left( t+\Delta t \right)=P_{N}\left( t \right)\left( 1-q\Delta t \right)\left( 1-Nc\Delta t \right)+P_{N-1}\left( t \right)q\Delta t\left( 1-\left( N-1 \right)c\Delta t \right)+P_{N+1}\left( t \right)\left( 1-q\Delta t \right)\left( N+1 \right)c\Delta t.$	(8)

$P_{N}\left( t+\Delta t \right)=P_{N}\left( t \right)\left( 1-\lambda\Delta t \right)\left( 1-nc\Delta t \right)+P_{N-1}\left( t \right)q\Delta t\left( 1-\left( n-1 \right)c\Delta t \right)+P_{N+1}\left( t \right)\left( 1-q\Delta t \right)nc\Delta t.$	(9)

$P_{N}\left( t+\Delta t \right)=P_{N}\left( t \right)\left( 1-q\Delta t \right)\left( 1-nc\Delta t \right)+P_{N-1}\left( t \right)q\Delta t\left( 1-nc\Delta t \right)+P_{N+1}\left( t \right)\left( 1-q\Delta t \right)nc\Delta t.$	(10)

Equations (9) and (10) become the same when higher orders of *∆t* are neglected, and so the transient equations become:

$P_{0}^{'}\left( t \right)=-qP_{0}\left( t \right)+cP_{1}\left( t \right).$	(11)

$P_{N}^{'}\left( t \right)=-\left( q+Nc \right)P_{N}\left( t \right)+qP_{N-1}\left( t \right)+\left( N+1 \right)cP_{N+1}\left( t \right).$	(12)

$P_{N}^{'}\left( t \right)=-\left( q+nc \right)P_{N}\left( t \right)+qP_{N-1}\left( t \right)+ncP_{N+1}\left( t \right).$	(13)

The steady state equations are obtained when the time derivatives are set to zero and equations are obtained as above only when *X=q/nc <1*. The solution of these steady state equations by induction are:

$P_{N}=\frac{{(nX)}^{N}}{N!} P_{0}$ for *N<n*.	(14)

and $P_{N}=\frac{{(nX)}^{N}}{n!n^{N-n}} P_{0}$	for *N ≥n*	(15)

*P_0_* can be found from,

$\sum_{N=0}^{\infty} P_{N}=\sum_{N=0}^{n-1} P_{N}+\sum_{N=n}^{\infty} P_{N}=1.$	(16)

Solving for *P_0_* gives,

$P_{0}=\frac{1}{\sum_{N=0}^{n-1} \left( \frac{{(nX)}^{N}}{N!} \right)+\frac{{(nX)}^{n}}{n!\left( 1-X \right)}}.$	(17)

From these expected number in the queue can be calculated as,

$E\left( m \right)=\sum_{N=n}^{\infty} (N-n)P_{N}=\sum_{N=n}^{\infty} \left( N-n \right)P_{0}\frac{{(nX)}^{N}}{n!n^{N-n}}.$	(18)

On simplification equation (18) becomes,

$E\left( m \right)=\frac{X}{n!}\frac{{(nX)}^{n}}{\left( 1-X \right)^{2}}P_{0}.$	(19)

From this, using the Little’s queueing formula [53], the average waiting time in the queue is,

$W_{q}=\frac{E\left( m \right)}{q}=\frac{X}{q n!}\frac{{(nX)}^{n}}{\left( 1-X \right)^{2}}\frac{1}{\sum_{N=0}^{n-1} \left( \frac{{(nX)}^{N}}{N!} \right)+\frac{{(nX)}^{n}}{n!\left( 1-X \right)}}.$	(20)

Equation (20) represents the *d_2_* of the delay formulation. Then, the average control delay is obtained by the addition of uniform and random delay, and it is expressed as:

$d=\frac{C\left( 1-\lambda\right)^{2}}{2\left( 1-\lambda X \right)}+\frac{X}{q n!}\frac{{(nX)}^{n}}{\left( 1-X \right)^{2}}\frac{1}{\sum_{N=0}^{n-1} \left( \frac{{(nX)}^{N}}{N!} \right)+\frac{{(nX)}^{n}}{n!\left( 1-X \right)}}$.	(21)

Equation (21) represents the delay expression for MCLF under-saturated traffic condition.”

A list of notations used in these equations and throughout the manuscript, along with their description, is provided in the Table given below. This table has been included in the “**Study site, data and preliminary analysis”** section of the manuscript.

**Table. List of notations**

| **Notation** | | **Description** |
| --- | --- | --- |
| **Signal control related variables** | | |
| *C* | Cycle length (sec) | |
| *R* | Effective red time for a phase (sec) | |
| *g* | Effective green time for a phase (sec) | |
| *λ* | Proportion of the effective green to cycle length (g/C) | |
| *q* | Traffic demand (vehicles/hour or PCE/hour) | |
| *s* | Saturation flow rate (vehicles/hour or PCE/hour) | |
| *c* | Capacity rate (vehicles/hour or PCE/hour), c=sg/C | |
| **Theoretical delay model variables** | | |
| *d* | Average delay on a single approach to an intersection (sec/vehicles or sec/PCE) | |
| *d_1_* | Uniform delay (sec/vehicles or sec/PCE) | |
| *d_2_* | Random delay (sec/vehicles or sec/PCE) | |
| *d_3_* | Initial queue delay (sec/vehicles or sec/PCE) | |
| *PF* | Progression Adjustment Factor | |
| *I* | Ratio of variance to mean count of vehicles /Upstream filtering/metering adjustment factor | |
| *T* | Analysis period duration (hour) | |
| *X_0_* | Degree of saturation below which the overflow delay is negligible in capacity guide models | |
| *Q_b_* | Initial queue at the start of analysis period T (vehicles) | |
| *u* | Delay parameter | |
| *k* | Incremental delay factor dependent on controller settings | |
| *X* | Degree of saturation, ratio of actual flow to capacity, X=q/c  Utilisation factor in the context of queueing theory. For a multi-server system,  $X$ =q/nµ= q/c | |
| **Queueing theory related variables** | | |
| *W_q_* | Average waiting time in the queue | |
| *n* | Number of service channels or number of virtual lanes | |
| *µ* | Discharge rate (vehicles/hour or PCE/hour) | |
| *N* | Number of entities in the system | |
| *P_o_* | Probability of zero entities in the system | |
| *P_N_* | Probability of N entities in the system | |
| *p* | Average processing time (s) | |
