# Supplementary material for: An analytical delay model for multi-class and lane-free traffic condition
Source: PLoS One. 2025 Feb 21;20(2):e0319325. doi: 10.1371/journal.pone.0319325 (PMC11845045; doi:10.1371/journal.pone.0319325)
Supplement: Attachment — Submitted filename: Response to reviews.docx [file pone.0319325.s003.docx]

**PONE-D-24-24169**

**An Analytical Delay model for Multi class and Lane free Traffic condition**

We thank the reviewers and the editor for their constructive comments. We have provided our responses below and the same have been incorporated in the revised manuscript appropriately.

**Academic editor**

**Comment 1:**

Please ensure that your manuscript meets PLOS ONE’s style requirements, including those for file naming. The PLOS ONE style templates can be found at <https://es.sonicurlprotection-tko.com/click?PV=2&MSGID=202409102239300160340&URLID=18&ESV=10.0.30.8013&IV=EC3522104D93790BD180B0EEC3FD6C25&TT=1726007974232&ESN=x%2Fbf%2FhvQwQUPhEXeVGmruMI6jhWkBQ7Ndy7gKzQip84%3D&KV=1536961729280&B64_ENCODED_URL=aHR0cHM6Ly9qb3VybmFscy5wbG9zLm9yZy9wbG9zb25lL3MvZmlsZT9pZD13alZnL1BMT1NPbmVfZm9ybWF0dGluZ19zYW1wbGVfbWFpbl9ib2R5LnBkZg&HK=B610C60AB030BFB99C3BF29F3923F4A1D78E34DC11FE710ECD96AF14676313CF> and

<https://es.sonicurlprotection-tko.com/click?PV=2&MSGID=202409102239300160340&URLID=17&ESV=10.0.30.8013&IV=D4C5285796F23814241E0C6D25BA7090&TT=1726007974232&ESN=wSB8QD2kWGn%2F%2F3Tb4RGJfkweZvspTakOxt6PtczblAA%3D&KV=1536961729280&B64_ENCODED_URL=aHR0cHM6Ly9qb3VybmFscy5wbG9zLm9yZy9wbG9zb25lL3MvZmlsZT9pZD1iYTYyL1BMT1NPbmVfZm9ybWF0dGluZ19zYW1wbGVfdGl0bGVfYXV0aG9yc19hZmZpbGlhdGlvbnMucGRm&HK=36D0A776BADA6DAD090AA1116D7368D98B9B3EBA14BFC77B8B4A6FB1EED93BE8>

**Response:** Thank you for your feedback and for allowing us to submit the revised version of the manuscript. We have revised and ensured that our manuscript now meets all of PLOS ONE’s style requirements, including the specific guidelines for file naming.

**Comment 2:**

In your Methods section, please provide additional information regarding the permits you obtained for the work. Please ensure you have included the full name of the authority that approved the field site access and, if no permits were required, a brief statement explaining why.

**Response:** Thank you for this comment. In India, the authority responsible for granting permission for video data collection at field sites is the police, and they granted the permission for video data collection. No additional permits were required for this study. Based on your suggestion, we have acknowledged their support in the Acknowledgments section as:

“Authors acknowledge CSIR-Central Road Research Institute (CRRI), New Delhi, India, for providing the traffic video for the study site 1. The authors acknowledge the Chennai traffic police for granting permission for video data collection for study site 2.”

**Comment 3:**

Thank you for stating the following financial disclosure:  "Ministry of Road Transport and Highways (MoRTH) " Please state what role the funders took in the study. If the funders had no role, please state: "

"The funders had no role in study design, data collection and analysis, decision to publish, or preparation of the manuscript." If this statement is not correct you must amend it as needed.  Please include this amended Role of Funder statement in your cover letter; we will change the online submission form on your behalf**.**

**Response:** Thank you for your comment regarding the financial disclosure. The Ministry of Road Transport and Highways (MoRTH) funded this study. However, the funders had no role in the study design, data collection and analysis, decision to publish, or preparation of the manuscript. We have included this statement in the cover letter and will rely on your team to update the online submission form accordingly.

**Comment 4:**

In the online submission form, you indicated that "Available upon request"

All PLOS journals now require all data underlying the findings described in their manuscript to be freely available to other researchers, either 1. In a public repository, 2. Within the manuscript itself, or 3. Uploaded as supplementary information.

This policy applies to all data except where public deposition would breach compliance with the protocol approved by your research ethics board. If your data cannot be made publicly available for ethical or legal reasons (e.g., public availability would compromise patient privacy), please explain your reasons on resubmission and your exemption request will be escalated for approval.

**Response:** Thank you for your feedback regarding data availability. In compliance with this, we have now provided the relevant data as supplementary information with our resubmission.

**Comment 5:**

We note that Figures 1(a) and 2(a) in your submission contain [map/satellite] images which may be copyrighted. All PLOS content is published under the Creative Commons Attribution License (CC BY 4.0), which means that the manuscript, images, and Supporting Information files will be freely available online, and any third party is permitted to access, download, copy, distribute, and use these materials in any way, even commercially, with proper attribution. For these reasons, we cannot publish previously copyrighted maps or satellite images created using proprietary data, such as Google software (Google Maps, Street View, and Earth)

**Response:** Thank you for your comment. Figures 1(a) and 2(a) are snapshots from OpenStreetMap, which is open data licensed under the [Open Data Commons Open Database License](https://opendatacommons.org/licenses/odbl/) (ODbL). The data is free to copy, distribute, transmit, and adapt. Hence, it adheres to the required licensing guidelines and ensures that all content can be freely accessed, distributed, and used as per the journal's policy.

**Reviewer #1**

This paper aims to propose a delay model for multi class and lane free traffic condition. Overall, I agree that a different delay model is needed under the new traffic operation mode, i.e., lane free traffic. My detailed comments are as follows:

**Comment 1:**

It is stated that “Key features include ... lane-free movement prevalent in Indian traffic.” Please clarify how to consider the lane-free movement?

**Response:** Thank you for this query. In this study, lane-free movement is addressed using the concept of virtual lanes, which is discussed in the section on the number of servers. To clarify this further, we have revised the section as follows:

“Virtual lane concept is utilised to find the number of servers occurring in the lane-free movement. The number of servers is based on the number of vehicles crossing the stop bar in parallel at any given moment. Since the composition of vehicles crossing the stop bar varies over time, this number fluctuates.

To establish an average value for the number of servers, the average number of parallel movements is considered at three different times during the queue dissipation process: (a) the onset of queue dissipation (5 seconds after the start of the green signal), (ii) the midway through the queue (at 30% of the green time), and (iii) upon completion of queue dissipation. It is assumed that the number of virtual lanes remains constant for a given road geometry, vehicle composition, traffic, and control conditions.”

**Comment 2:**

Some recent studies have described the two-dimensional movement. E.g., Developing an optimal intersection control system for automated, connected vehicles, IEEE T-ITS, 2018; Unprotected left-turn behaviour model capturing path variations at intersections, IEEE T-ITS, 2023; A behaviourally underpinned approach for two-dimensional vehicular trajectory reconstruction with constrained optimal control, TR-Part C, 2024. However, the authors did not mention them at all.

**Response:** Thank you for your comments and for suggesting these papers for review. The authors have reviewed the suggested papers, and the details are provided below.

1. Y Bichiou, H A Rakha. Developing an Optimal Intersection Control System for Automated Connected Vehicles. IEEE Transactions on Intelligent Transportation Systems, 2019. 20(5).

The authors have carefully reviewed the suggested paper and acknowledge that while they focus on connected vehicles and employ a car-following model tailored to autonomous systems, our study centres on conventional vehicles. As such, we recognize that the two-dimensional movement characteristics of conventional vehicles differ significantly from those of connected vehicles, which was a core consideration in our approach.

2. J Zhao, VL Knoop, J Sun, Z Ma, M Wang. Unprotected Left-Turn Behavior Model Capturing Path Variations at Intersections. IEEE Transactions on Intelligent Transportation Systems, 2023. 24(9):9016-9030.

This study investigates the overall path dispersion of unprotected left turns and opposing through movements at intersections. The path dispersion reflects the operational features of traffic flow and is influenced by driving behaviour, arrival flow patterns, layout design, and the traffic control and management scheme. We have incorporated the findings from this study into the literature review section of our paper, as follows:

“Specific characteristics of MCLF traffic with a wide variety of vehicle classes with lane-free movement may need to be incorporated in the model for it to perform well. One of the main characteristics is the smaller vehicles squeezing through the space between the larger ones, flouting the first-in, first-out (FIFO) queueing rule. A lot of parallel movements occur both within and across lanes as a result of lane-free movement [36]. The movement of vehicles can be affected by factors such as geometric design, signal control settings, driver behaviour and traffic conditions [37].”

3. Jing Zhao, Ruoming Ma, Meng Wang: A behaviourally underpinned approach for two-dimensional vehicular trajectory reconstruction with constrained optimal control, Transportation Research Part C: Emerging Technologies, 2024. 159, Article 104489.

This paper discusses the trajectory reconstruction with constraint variables, including velocity, longitudinal acceleration, jerk, curvature, lateral acceleration, and change of curvature. However, our study focuses on a delay model based on queuing theory principles, which does not rely on vehicle trajectories. As such, the variables considered in the referenced model are not included in our approach, as our primary objective was to develop a model for delay estimation.

**Comment 3:**

Model validation was not conducted using actual data, but rather simulation data was used as ground truth. Why can simulation be considered as ground truth?

**Response:** Thank you for your insightful comment. In response to your comment, we have added a new sub-section on “Model validation using field data” in the model validation section as follows:

**“Model validation and results**

The developed model was validated using the delay values calculated using the field data of Mhalgi Nagar Square Intersection, Nagpur and Kaiveli Intersection, Chennai. Additionally, the performance of the model is evaluated using the various scenarios generated in the calibrated and validated VISSIM network of the Mhalgi Nagar Square intersection.

**Model validation using field data**

The delay values of each cycle obtained from the field data were compared with the delay estimates from the proposed delay model. The comparative assessment of average approach delays for Mhalgi nagar and Kaiveli are given below.

Cycle-by-cycle comparison of delay estimates of Proposed model with field data of Mhalgi nagar, Nagpur site

Cycle-by-cycle comparison of delay estimates of Proposed model with field data of Kaiveli, Chennai site

From these figures, it is evident that the proposed delay model effectively captures the actual delay values under both field conditions. The average Mean Absolute Percentage Error (MAPE) for the delay values at Mhalgi Nagar, Nagpur, was 14%. Among the 10 observed cycles at Kaiveli, 2 cycles were over-saturated, where the applicability of the steady-state stochastic model is reported to be limited. Hence, the comparative results for the remaining 8 cycles are presented in Figure 12, with each cycle exhibiting good accuracy between the field-observed and model-predicted values. The average MAPE for these cycles was 12%.”

Furthermore, the previously submitted manuscript included model validation results for field conditions simulated in VISSIM, which has now been incorporated as one scenario in the sensitivity analysis. As a result, the sensitivity analysis has been updated with model validation results for varying X values, ranging from 0.5 to 0.9, with 0.5 representing the field conditions developed in VISSIM. The revised section is as follows:

**“Model validation using simulated data and sensitivity analysis**

To better understand the performance of the proposed delay model at other *X* values, scenarios with *X* values ranging from 0.5 to 0.9 were generated in the Mhalgi nagar VISSIM network. The delay results obtained from the proposed delay model were compared against those obtained from VISSIM, Webster’s delay model [22], Akcelik delay model [31], HCM model [35] and Indo-HCM delay model [41]. The MAPE values of each delay model compared to the VISSIM delay were calculated and illustrated in Fig 13.

MAPE values of delay models compared with VISSIM delay

In all scenarios considered, the proposed delay model demonstrated superior performance as evidenced by lower MAPE values. The MAPE value of the proposed delay model decreased from 0.5 to 0.9, indicating its coherence to actual delay at higher *X* values.”

In addition to this, details on the field estimation of delay have been added to the section “Study site, data, and preliminary analysis”.

“For the validation, the average vehicular delay experienced during this time was also extracted. To achieve this, the time at which individual vehicles came to a stop and the time they started moving for each signal cycle were meticulously extracted for one approach. The acceleration and deceleration delays were calculated by considering their respective rates [42]. These were added to the stopped delay and then were used for validation.”

**Comment 4:**

Are the virtual lane numbers dynamically changing? According to the author's assumption, the virtual lane numbers seem to vary under different circumstances. How were they handled during model development?

**Response:** Thank you for your thoughtful comment. The number of virtual lanes is considered in this study, as a constant for a given vehicle composition and road geometry. The virtual lane numbers may vary based on vehicle composition. To address this, the average number of virtual lanes is calculated by considering three key instances during queue dissipation: at the beginning, mid-point, and end of the queue. To address this comment, we have revised the explanation of the virtual lane concept as follows:

“The smaller vehicles often squeeze through the space between the larger ones, flouting the FIFO queueing rule. Hence, the number of lanes marked on the road will not be equal to the number of service channels. Virtual lane concept is utilised to find the number of servers occurring in the lane-free movement. The number of servers is based on the number of vehicles crossing the stop bar in parallel at any given moment. Since the composition of vehicles crossing the stop bar varies over time, this number fluctuates.

To establish an average value for the number of servers, the average number of parallel movements is considered at three different times during the queue dissipation process: (a) the onset of queue dissipation (5 seconds after the start of the green signal), (ii) the midway through the queue (at 30% of the green time), and (iii) upon completion of queue dissipation [40]. It is assumed that the number of virtual lanes remains constant for a given road geometry, vehicle composition, traffic, and control conditions. Table 5 shows the example calculation of virtual lanes for the Kaiveli study site. The actual number of lanes was 3, even though the effective number of service channels increased to 7 due to parallel movements. Similarly, the number of virtual lanes was calculated at the Mhalgi nagar intersection, where 3 lanes were functioning as equivalent to 5 service channels.”

**Comment 5:**

How were the parameters in the theoretical model calibrated?

**Response:** Thank you for this query. In the theoretical delay model, the parameters are related to traffic control, traffic volume, and road geometry. Among these, the only constant parameter is the number of virtual lanes (n), which depends on the geometry and vehicle composition. The average control delay at each study site is calculated using traffic volume and signal control data specific to that site. As a result, this theoretical delay model does not require a particular data set for calibration. Instead, it can be applied directly based on the traffic conditions and control data available for each location.

**Comment 6:**

How are the datasets for model calibration and model testing distinguished?

**Response:** Thank you for your question. The developed theoretical model does not require any datasets for calibration. Instead, the collected field data is utilized for model validation. This distinction allows us to assess the model's accuracy and reliability using real-world traffic conditions without the need for preliminary calibration datasets.

**Reviewer #2**

**Comment 1:**

Literature Review is very short. A significant number of papers discussing lane-free roads should be included in this section. Please add and provide an explanation of the articles listed below:

1. Papageorgiou, M., Mountakis, K.S., Karafyllis, I., Papamichail, I., Wang, Y.: Lane-free artificial-fluid concept for vehicular traffic, Proceedings of the IEEE 109 (2021), pp. 114-121.
2. Malekzadeh, M., Papamichail, I., Papageorgiou, M., Bogenberger, K.: Optimal internal boundary control of lane-free automated vehicle traffic, Transportation Research Part C 126 (2021), Article 103060.
3. Malekzadeh, M., Papamichail, I., Papageorgiou, M.: Linear-Quadratic regulators for internal boundary control of lane-free automated vehicle traffic, Control Engineering Practice 115 (2021), Article 104912.
4. Karafyllis, I., Theodosis, D., Papageorgiou, M.: Analysis and control of a non-local PDE traffic flow model, International Journal of Control 95 (2022), pp. 660-678.
5. Karafyllis, I., Theodosis, D., Papageorgiou, M.: Lyapunov-based two-dimensional cruise control of autonomous vehicles on lane-free roads, Automatica 145 (2022), Article 110517.
6. Karafyllis, I., Theodosis, D., Papageorgiou, M.: Constructing artificial traffic fluids by designing cruise controllers, Systems & Control Letters 167 (2022), Article 105317.
7. Karafyllis, I., Theodosis, D., Papageorgiou, M.: Nonlinear adaptive cruise control of vehicular platoons, International Journal of Control 96 (2023), pp. 147-169. - -
8. Malekzadeh, M., Yanumula, V.K., Papamichail, I., Papageorgiou, M.: Overlapping internal boundary control of lane-free automated vehicle traffic, Control Engineering Practice 133 (2023), Article 105435.
9. Yanumula, V.K., Typaldos, P., Troullinos, D., Malekzadeh, M., Papamichail, I., Papageorgiou, M.: Optimal trajectory planning for connected and automated vehicles in lane-free traffic with vehicle nudging, IEEE Transactions on Intelligent Vehicle 8 (2023), pp. 2385-2399.
10. Karafyllis, I., Papageorgiou, M.: A particle method for 1-D compressible fluid flow, Studies in Applied Mathematics 151 (2023), pp. 1282–1331.
11. Malekzadeh, M., Troullinos, D., Papamichail, I., Papageorgiou, M., Bogenberger, K.: Internal boundary control in lane-free automated vehicle traffic: Comparison of approaches via microscopic simulation, Transportation Research Part C 158 (2024), Article 104456.
12. Naderi, M., Papageorgiou, M., Troullinos, D., Karafyllis, I., Papamichail, I.: Controlling automated vehicles on large lane-free roundabouts, IEEE Transactions on Intelligent Vehicle 9 (2024), pp. 3061-3074.
13. Jin, X., Yu, X., Hu, Y., Wang, Y., Papageorgiou, M., Papamichail, I., Malekzadeh, M.: Integrated control of internal boundary and ramp inflows for lane-free traffic of automated vehicles on freeways. 25th IEEE International Conference on Intelligent Transportation Systems (ITSC 2022), Macau, China, October 8-12, 2022, pp. 1234-1239
14. Malekzadeh, M., Manolis, D., Papamichail, I., Papageorgiou, M.: Empirical investigation of properties of lane-free automated vehicle traffic. 25th IEEE International Conference on Intelligent Transportation Systems (ITSC 2022), Macau, China, October 8-12, 2022, pp. 2393-2400.
15. Papamichail, I., Schoenn-Anchling, N., Malekzadeh, M., Markantonakis, V., Papageorgiou, M.: Macroscopic traffic flow model calibration for lane-free automated vehicle traffic. 26th IEEE International Conference on Intelligent Transportation Systems (ITSC 2023), Bilbao, Bizkaia, Spain, September 24-28, 2023, pp. 3485-3492

**Response:** Thank you for your insightful comment on the literature review. Based on your suggestion, the authors have reviewed the above-mentioned papers on lane-free movement. However, studies [1-6, 8-9, 11-15] address the lane-free movement of Connected Automated Vehicles (CAV) using the concept of Internal Boundary Control (IBC). This concept entails virtually shifting the internal boundary that separates two directions of traffic, enabling flexible real-time sharing of the total road capacity between them based on traffic conditions. However, our study specifically focuses on the lane-free movement of vehicles in a single direction. It does not involve sharing the approach width between two directions but rather explores the utilization of lanes designated for one-directional movement without adhering to the traditional lane markings. The other referenced paper [7] discusses the design of nonlinear adaptive cruise controllers for vehicular platoons operating on open or ring roads, whereas our study focuses on the estimation of signalized intersection delay. Paper [10] addresses the convergent approximations of weak solutions to the Navier-Stokes equations for the 1-D flow of a viscous compressible fluid, which is not relevant to the focus of our study. Hence, these are not added to the literature review section. However, based on the comment that the literature review is short, more relevant papers were identified and added to the literature review as follows.

“**Deterministic delay models**

The deterministic queuing models can accurately estimate delay at signalized intersections, where green interval serve a greater number of vehicles than the number of arrivals per cycle [13]. The first deterministic delay model was introduced at the 7^th^ annual meeting of the Highway Research Board [14]. The area between the cumulative arrivals and departures are considered as the deterministic component of delay and is estimated according to the following assumptions: i) zero initial queue at the start of the green phase, ii) uniform arrival pattern at the arrival flow rate (*q*) throughout the cycle iii) uniform departure pattern at the saturation flow rate (*S*) during queue discharge, and at the arrival rate when the queue vanishes, and iv) arrivals do not exceed the signal capacity, defined as the product of approach saturation flow rate (*S*) and its effective green to cycle ratio (*g/C*). Newel [15] employed the continuum model, using *A(t)* and *D(t)* to represent cumulative arrivals and departures at any time *t*. The arrival curve is shifted by the free flow travel time to get the virtual arrival curve. The area between the virtual arrival and departure curve for a cycle gives the total delay per cycle. May and Keller [16] developed a delay model with a deterministic assumption for over-saturated conditions. In reality, the randomness of traffic may cause fluctuations, rendering the deterministic assumption invalid.

**Steady state stochastic models**

The deterministic models rely on the assumption of uniform arrivals, while stochastic delay models aim to incorporate the variability associated with vehicle arrivals [17]. Beckmann et al. [18] proposed a stochastic model for vehicle delay with binomial arrivals and uniform departures. Newell [19] applied the Beckmann model to various arrival rates and demonstrated that binomial arrivals, which ensure a minimum spacing between vehicles, prevent infinite delays as *q/s* approaches unity. Although this model provided higher delay estimates than those based on deterministic arrivals, it still underestimated the observed delay values [20]. Later, vehicle arrivals at intersections were modelled as a Poisson process [21]. The model incorporated a random delay term and a uniform delay term to calculate the total delay per vehicle. Webster [22] modified the above M/D/1 model by adding empirical terms to Kendall’s model. It is comprised of three components. The first component estimates delay assuming a uniform arrival rate, while the second term accommodates the randomness in arrivals, known as the "random delay", which is derived for a Poisson arrival process and services at a constant rate equivalent to signal capacity. The third component, calibrated through simulation experiments, is an adjustment factor and typically in the range of 5-15 percent of the first two components.

Subsequent to Webster's work, several other stochastic models emerged [15,23–25]. Miller [23] developed a delay model based on a general arrival process and a deterministic service process, introducing the variance-to-mean ratio (*I*) to account for the random nature of arrivals. Miller's model gave better results compared to Webster’s model when the value of *I* is greater than one [15,20]. Later, Hutchinson [26] suggested modifications to the random delay term of Webster’s model by including the *I* ratio, resulting in more accurate delay estimates than Webster’s model. In comparison to binomial and uniform arrival types, the Poisson assumption yields better estimates when the degree of saturation is less [20]. However, the limitation of the Poisson assumption is that it permits for small time headway between arrivals, which can lead to infinite queue lengths and delays when the degree of saturation approaches unity [19]. These models share common basic assumptions. They assume the departure headways follow a known distribution with a constant mean. Furthermore, it is assumed that the system remains under-saturated throughout the analysis period despite temporary oversaturation due to the variability in arrivals. Finally, it is presumed that the system has been operational for a duration sufficient to stabilise into a steady state.

However, in reality, the assumptions of steady-state models are compromised when the flow rate fluctuates due to randomness in arrivals, as the stochastic equilibrium cannot be reached. Thus, there is a necessity for developing delay models taking into account these unique features, and is addressed in this study.

**Time dependent delay models**

Several attempts have been made to address the limiting assumption of steady-state conditions. One of the best approaches is to approximate time dependent arrival profile by some mathematical function and calculate corresponding delay. This method connects the steady-state stochastic delay model with the deterministic over-saturation model. Catling [27] and Akcelik [28] introduced the coordinate transformation technique to develop such a model. This technique connects the steady-state stochastic delay models to a deterministic over-saturation delay developed by May and Keller [16]. However, the deterministic oversaturation delay model overlooked initial queue delay, resulting in Catling and Akcelik's model also failing to capture this aspect. Kimber and Hollis [29] introduced a time-dependent delay model based on the assumption of random arrivals and general departures with one server system. The capacity guide delay models currently utilised in the United States [30], Australia [31], and Canada [32] are examples of time-dependent delay models. Akcelik [33] and Burrow [34] proposed generalised delay models from which the above models could be obtained by substituting suitable parameter values. Akcelik [33] demonstrated that the HCM 1985 [30] model overestimated the delay in over-saturated scenarios due to the *X^2^* term in the overflow delay component of the model. Akgungor and Bullen [10] recommended replacing *X^2^* with *X^n^* and also derived a non-linear function to calculate *n*. Fambro and Rouphail [9] suggested incorporating analysis period *T* in the HCM delay model and also proposed a third term (d_3_) to accommodate initial queue delays. The suggestions were adopted in the HCM 2000 [35] model.”

**Comment 2:**

The quality of the figures is not good. Please improve it.

**Response:** The authors thank the reviewer for this feedback regarding the quality of the figures. The resolution and clarity of all figures are improved in the revised manuscript.

**Comment 3:**

Please explain more about the equations.

**Response:** Based on this suggestion, additional explanations of the equations are added, as outlined below:

“A multi-channel queueing process is considered as the best way to represent the queue formation under MCLF traffic conditions. As the arrivals and departures are stochastic under MCLF traffic condition, the system is modelled as M/M/n system, which follows Poisson arrivals and departure with *n* service channels. The corresponding random delay term (*d2*) is derived based on this.

The basic derivation starts with the concept of birth-death process. One of the basic assumptions made in earlier studies [39] is the system is always operating at its full capacity. This assumption is not realistic for signalized intersection, as discharge occur only during green signals. Consequently, capacity is a more appropriate term for describing departure from a signalized intersection. Using this concept, for a multiple channel queueing system, there are four conditions at time *t* which must be considered in the formulation of transient equations, which are:

1. The system is empty with no entities (*N*=0).
2. The number of entities is less than the number parallel servers (*N<n*)*.*
3. The system with number of entities and number of parallel servers are same (*N=n*).
4. The number of entities is higher than the number of parallel servers (*N>n*).

Let $P_{0}\left( t+\Delta t \right)$ and $P_{N}\left( t+\Delta t \right)$ be the probability that the queueing system has zero and *N* entities at time $t+\Delta t$ . There are three ways in which the system could have reached this state, assuming $\Delta t$ is so small that only one unit could have entered or departed.

a) The system did not change from time t to $t+\Delta t$
b) The system was in state *N-1* at *t*, and one arrived in $\Delta t$
c) The system was in state *N+1* at *t*, and one departed in $\Delta t$

Since the probability of one arrival in $\Delta t$ equals $q\Delta t$ and the probability of one departure in $\Delta t$ is $c\Delta t$, the corresponding probabilities of zero arrivals and zero departures are *1 -* $q\Delta t$ and *1-*$c\Delta t$. If higher orders of $\Delta t$ are neglected, the mathematical description of the three ways for the system to reach *N* and zero entities in $t+\Delta t$ of the above-stated four conditions of multiple channel queueing system are:

$P_{0}\left( t+\Delta t \right)=P_{0}\left( t \right)\left( 1-q\Delta t \right)+P_{1}\left( t \right)\left( 1-q\Delta t \right)c\Delta t.$	(7)

$P_{N}\left( t+\Delta t \right)=P_{N}\left( t \right)\left( 1-q\Delta t \right)\left( 1-Nc\Delta t \right)+P_{N-1}\left( t \right)q\Delta t\left( 1-\left( N-1 \right)c\Delta t \right)+P_{N+1}\left( t \right)\left( 1-q\Delta t \right)\left( N+1 \right)c\Delta t.$	(8)

$P_{N}\left( t+\Delta t \right)=P_{N}\left( t \right)\left( 1-\lambda\Delta t \right)\left( 1-nc\Delta t \right)+P_{N-1}\left( t \right)q\Delta t\left( 1-\left( n-1 \right)c\Delta t \right)+P_{N+1}\left( t \right)\left( 1-q\Delta t \right)nc\Delta t.$	(9)

$P_{N}\left( t+\Delta t \right)=P_{N}\left( t \right)\left( 1-q\Delta t \right)\left( 1-nc\Delta t \right)+P_{N-1}\left( t \right)q\Delta t\left( 1-nc\Delta t \right)+P_{N+1}\left( t \right)\left( 1-q\Delta t \right)nc\Delta t.$	(10)

Equations (9) and (10) become the same when higher orders of *∆t* are neglected, and so the transient equations become:

$P_{0}^{'}\left( t \right)=-qP_{0}\left( t \right)+cP_{1}\left( t \right).$	(11)

$P_{N}^{'}\left( t \right)=-\left( q+Nc \right)P_{N}\left( t \right)+qP_{N-1}\left( t \right)+\left( N+1 \right)cP_{N+1}\left( t \right).$	(12)

$P_{N}^{'}\left( t \right)=-\left( q+nc \right)P_{N}\left( t \right)+qP_{N-1}\left( t \right)+ncP_{N+1}\left( t \right).$	(13)

The steady state equations are obtained when the time derivatives are set to zero and equations are obtained as above only when *X=q/nc <1*. The solution of these steady state equations by induction are:

$P_{N}=\frac{{(nX)}^{N}}{N!} P_{0}$ for *N<n*.	(14)

and $P_{N}=\frac{{(nX)}^{N}}{n!n^{N-n}} P_{0}$	for *N ≥n*	(15)

*P_0_* can be found from,

$\sum_{N=0}^{\infty} P_{N}=\sum_{N=0}^{n-1} P_{N}+\sum_{N=n}^{\infty} P_{N}=1.$	(16)

Solving for *P_0_* gives,

$P_{0}=\frac{1}{\sum_{N=0}^{n-1} \left( \frac{{(nX)}^{N}}{N!} \right)+\frac{{(nX)}^{n}}{n!\left( 1-X \right)}}.$	(17)

From these expected number in the queue can be calculated as,

$E\left( m \right)=\sum_{N=n}^{\infty} (N-n)P_{N}=\sum_{N=n}^{\infty} \left( N-n \right)P_{0}\frac{{(nX)}^{N}}{n!n^{N-n}}.$	(18)

On simplification, equation (18) becomes:

$E\left( m \right)=\frac{X}{n!}\frac{{(nX)}^{n}}{\left( 1-X \right)^{2}}P_{0}.$	(19)

From this, using the Little’s queueing formula [51], the average waiting time in the queue is,

$W_{q}=\frac{E\left( m \right)}{q}=\frac{X}{q n!}\frac{{(nX)}^{n}}{\left( 1-X \right)^{2}}\frac{1}{\sum_{N=0}^{n-1} \left( \frac{{(nX)}^{N}}{N!} \right)+\frac{{(nX)}^{n}}{n!\left( 1-X \right)}}.$	(20)

Equation (20) represents the *d_2_* of the delay formulation. Then, the average control delay is obtained by the addition of uniform and random delay, and it is expressed as:

$d=\frac{C\left( 1-\lambda\right)^{2}}{2\left( 1-\lambda X \right)}+\frac{X}{q n!}\frac{{(nX)}^{n}}{\left( 1-X \right)^{2}}\frac{1}{\sum_{N=0}^{n-1} \left( \frac{{(nX)}^{N}}{N!} \right)+\frac{{(nX)}^{n}}{n!\left( 1-X \right)}}$.	(21)

Equation (21) represents the delay expression for MCLF under-saturated traffic condition. At the study sites, Mhalgi nagar and Kaiveli intersection, the number of parallel serves was 5 and 7 respectively. By substituting the respective values of *n* into Equation (21), the delay equation for Mhalgi nagar and Kaiveli site were obtained and are given in (22) and (23) respectively.

$d=\frac{C\left( 1-\lambda\right)^{2}}{2\left( 1-\lambda X \right)}+\frac{625X^{6}}{q(1-X)(24+96X+180X^{2}+200X^{3}+125X^{4})}$.	(22)

$d=\frac{C\left( 1-\lambda\right)^{2}}{2\left( 1-\lambda X \right)}+\frac{117649X^{8}}{q(1-X)(720+4320X+12600X^{2}+23520X^{3}+30870X^{4}+28812X^{5}+16807X^{6})}.$	(23)

It is essential to validate this model to ensure its accuracy and applicability in the real-world traffic scenario, and is explained in the following section.”
